# Label-Free Delineation of Brain Tumors by Coherent Anti-Stokes Raman Scattering Microscopy in an Orthotopic Mouse Model and Human Glioblastoma

**DOI:** 10.1371/journal.pone.0107115

**Published:** 2014-09-08

**Authors:** Ortrud Uckermann, Roberta Galli, Sandra Tamosaityte, Elke Leipnitz, Kathrin D. Geiger, Gabriele Schackert, Edmund Koch, Gerald Steiner, Matthias Kirsch

**Affiliations:** 1 Neurosurgery, University Hospital Carl Gustav Carus, Technische Universität Dresden, Dresden, Germany; 2 Clinical Sensoring and Monitoring, Department of Anesthesiology and Intensive Care Medicine, Faculty of Medicine Carl Gustav Carus, Technische Universität Dresden, Dresden, Germany; 3 Neuropathology, University Hospital Carl Gustav Carus, Technische Universität Dresden, Dresden, Germany; Cedars-Sinai Medical Center, United States of America

## Abstract

**Background:**

Coherent anti-Stokes Raman scattering (CARS) microscopy provides fine resolution imaging and displays morphochemical properties of unstained tissue. Here, we evaluated this technique to delineate and identify brain tumors.

**Methods:**

Different human tumors (glioblastoma, brain metastases of melanoma and breast cancer) were induced in an orthotopic mouse model. Cryosections were investigated by CARS imaging tuned to probe C-H molecular vibrations, thereby addressing the lipid content of the sample. Raman microspectroscopy was used as reference. Histopathology provided information about the tumor's localization, cell proliferation and vascularization.

**Results:**

The morphochemical contrast of CARS images enabled identifying brain tumors irrespective of the tumor type and properties: All tumors were characterized by a lower CARS signal intensity than the normal parenchyma. On this basis, tumor borders and infiltrations could be identified with cellular resolution. Quantitative analysis revealed that the tumor-related reduction of CARS signal intensity was more pronounced in glioblastoma than in metastases. Raman spectroscopy enabled relating the CARS intensity variation to the decline of total lipid content in the tumors. The analysis of the immunohistochemical stainings revealed no correlation between tumor-induced cytological changes and the extent of CARS signal intensity reductions. The results were confirmed on samples of human glioblastoma.

**Conclusions:**

CARS imaging enables label-free, rapid and objective identification of primary and secondary brain tumors. Therefore, it is a potential tool for diagnostic neuropathology as well as for intraoperative tumor delineation.

## Introduction

During surgery, the identification of tumor borders is a challenge due to the infiltrative nature of most brain tumor types. Glioblastoma multiforme (GBM), the most malignant primary brain tumor in adults, infiltrates normal brain tissue beyond the area of enhancement on MRI [1] and the infiltration cannot be readily recognized during the surgery. MR imaging represents the clinical gold standard but is not able to delineate fine structural differences. GBM accumulates orally administered 5-aminolevulinic acid which is metabolized to fluorescent compounds which can be used for intraoperative tumor visualization. The utilization of 5-aminolevulinic acid induced fluorescence is widely accepted for GBM resection, but in discussion of other tumor types [2]. Intraoperative tumor diagnosis requires tissue removal, processing, staining, and evaluation by a trained neuropathologist [3]. The final diagnosis of the tumor is obtained days after surgery by performing multiple immunohistochemical stainings on tissue sections. Present research focuses on the development of advanced, label-free techniques for objective, in situ brain cancer pathology assessment and new tools for intraoperative identification of tumor margins.

Coherent anti-Stokes Raman scattering (CARS) microscopy was evaluated to be a fast label-free imaging technique for these purposes. CARS is a nonlinear variant of Raman spectroscopy [4]. It is based on resonant excitation of the anti-Stokes field in a nonlinear process through irradiation with two ultra-short laser beams whose frequency difference is tuned to match the energy of a molecular oscillation [5]. Because CARS is a nonlinear process, the intensity of the signal is proportional to the square of the number of molecular bonds [6]. This implies that CARS imaging is inherently sensitive to small variations, and that it is more sensitive than spontaneous Raman spectroscopy, which is a linear process [6]. Most commonly, CARS imaging addresses the methylene Raman band at 2850 cm^−1^, and mainly probes the distribution of lipids [5]–[8] displaying the brain structures [9]–[11]. Because CARS microscopy is a label-free technique that directly targets the biochemical composition of the tissue and additionally provides morphological information at high speed, it is ideally suited for unprocessed ex vivo samples as well as for in vivo applications [12], [13].

The feasibility of CARS microscopy for tissue diagnosis using unstained brain tissue was first demonstrated by Evans et al. in 2007 [9]. CARS imaging of a fresh unfixed ex vivo mouse brain specimen was used to discern astrocytoma from normal brain tissue. Meyer et al. investigated a human sample of a brain metastasis from lung carcinoma [10] and applied image processing for cell nuclei detection in CARS images of a squamous cell carcinoma brain metastasis [14]. Comparative studies that include multiple brain tumor samples and that address tumor properties are lacking.

Here, we present detailed analyzes of CARS images on an extended set of different primary and secondary brain tumors grown in a mouse model, with the aim to understand whether the technique can: i) visualize and localize brain tumors with different properties, ii) discern tumor borders and tumor infiltrates, iii) supply useful information for the neuropathological assessment. CARS images were directly compared to immunohistochemical stainings in order to evaluate the relationship of CARS signal and tumor properties. Additionally, the CARS signal intensities generated in normal tissue as well as various tumors were evaluated and compared with reference Raman measurements.

## Methods

### Ethics statement

Animal experiments were conducted in accordance with the institutional and national guidelines in full agreement with the European Union directives. They were approved by the animal welfare committee of Saxony, Germany (Regierungspräsidium Dresden, AZ: 24-9168.11-1/2011-39). All surgery was performed under ketamine–xylazine anesthesia, and all efforts were made to minimize suffering. Mice were sacrificed by cervical dislocation.

Human brain tumor tissue was obtained during routine tumor surgery. The patients gave written consent and the study was approved by the ethics committee at University Hospital Carl Gustav Carus, Technische Universität Dresden, Germany (EK 323122008).

### Animal experiments

Experiments were performed on 16 immune deficient female nude mice NMRI nu/nu (Experimental Center, University Hospital Dresden, Germany). Animals were kept under pathogen-free conditions in a 12 h∶12 h light-dark cycle and received food and water ad libitum. Brain tumors were induced by stereotactic implantation of human tumor cell lines into the brain parenchyma as described previously [15]. Eight animals were implanted with GBM cells (U87MG), four animals with melanoma (A375) cells, and four animals with breast cancer cells (MCF-7). Within three weeks, the tumor-bearing brains were removed, embedded in tissue freezing medium (Leica, Nussloch, Germany), and frozen on dry ice.

### Human brain tissue

Human brain tumor samples (glioblastoma, n = 6) were snap frozen in liquid nitrogen and stored at −80°C. For sample preparation, tissue was embedded in tissue freezing medium.

### Sample preparation and histology

Cryosections of 16 µm thickness were prepared on glass slides and imaged with CARS microscopy. The measured sections as well as consecutive and non-consecutive sections were subjected to standard hematoxylin and eosin (H&E) staining and immunohistochemistry or placed on calcium fluoride slides in order to perform reference Raman spectroscopy.

For immunochistochemistry, the sections were blocked with 0.1% bovine serum albumine and 3% normal goat serum in 0.3% TritonX for 1 h and probed with the primary antibody for 1 h. After washing with PBS, the sections were incubated with the secondary antibody followed by colorimetric detection of the antibody signal. In detail, anti-CD31 (1∶500, provided by Prof. Breier, Pathology, University Hospital Dresden, Germany) was detected using Vectastain Elite ABC Kit Rat IgG (Vector Laboratories Inc., Burlingame, CA, USA) followed by AEC-kit (Vector laboratories). The sections were counterstained with eosin and coverslipped with aquatex (Merk, Darmstadt, Germany). Anti-Ki67 (1∶500, Leica Biosystems GmbH, Nußloch, Germany) was used with prior heat antigen retrieval in citrate buffer and in combination with histofine simple stain MAX Po (Nichirei Biosciences Inc., Tokyo, Japan) followed by histogreen (LINARIS Biologische Produkte GmbH, Dossenheim, Germany) detection, counterstaining with nuclear fast red, and mounting with DePeX (SERVA Electrophoresis GmbH, Heidelberg, Germany).

### Quantification of proliferation and vascularization

The microvessel density was determined at 200-fold magnification in three representative fields of view [16]. Additionally, the morphology of microvessels was evaluated. The thickness of the structures and the pattern of the vessels were analyzed and transformed to a scoring system: 0 = very fine, regular; 1 = fine, regular; 2 = coarse, regular; 3 = coarse, irregular [16].

To evaluate the proliferation index within the tumors, the proportion of Ki67 positive nuclei was determined in three fields of view within the neoplastic tissue at 400-fold magnification. All cells displaying a positive signal in more than 50% of their nuclei were included. In normal brain parenchyma, no Ki67-positive cells were detected.

The cross-sectional area of nuclei was measured using images acquired at 200-fold magnification in three (104×104) µm^2^ large fields-of-view using Fiji software [17]. The quantification was done in a blinded fashion.

### CARS imaging

**Figure 1:**
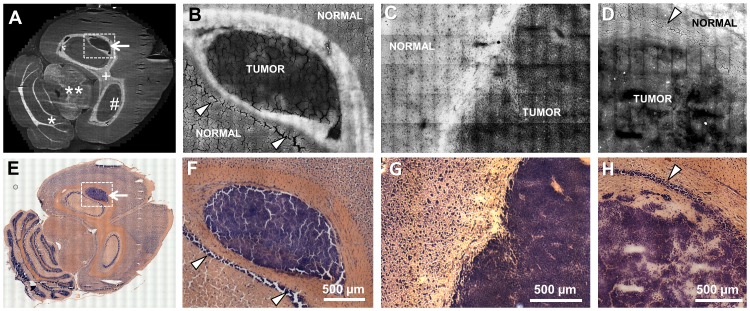
Imaging of experimental human glioblastoma and brain metastases in a mouse model. **A**: CARS image of a whole mouse brain section with an experimental human U87MG glioblastoma; white matter tracts appear brighter in the CARS image, while the tumor (arrow) is darker that the normal brain tissue. # hippocamus, + corpus callosum, **mesencephalon, *cerebellum **B**: Magnified CARS image of the area indicated in A; the difference in the CARS signal intensity between the neoplastic tissue and the surrounding white matter enables discerning the tumor border. **C**: CARS image of a breast cancer metastasis (induced by MCF-7 cells) in a mouse brain. **D**: CARS image of a metastasis of melanoma (induced by A375 cells) in the hippocampal region of a mouse brain. **E–F**: H&E stainings of consecutive sections corresponding to A–D. The arrowheads indicate nuclei-rich layers in gray matter of the hippocampus.

The system used for CARS imaging was described previously [18]. In the present study, the excitation beams were focused with a C-Apochromat 32×/0.85NA objective. An individual field-of-view image was acquired within 300 ms. The acquisition of large areas was performed with a tiling procedure; z-stacks were acquired in order to compensate for the lack of planarity of samples and followed by maximum intensity projections to obtain the final images. Acquisition times for the CARS overview images ranged from ∼5 min (Fig. 3A) to ∼30 min (Fig. 1A).

### CARS signal quantification

The intensity values of the CARS signal in the images were evaluated using the Fiji software [17]. Regions of interest (ROIs) of the tissue (gray matter and tumor) were manually chosen and the average pixel intensity in the ROIs was calculated.

### Raman microspectroscopy

Raman spectra acquisition and preprocessing were performed as described previously [18]. Spectral line maps with a step size of 17 µm were collected by sequentially exciting 50 to 70 sample points spanning over regions of tumor and normal tissue. For each position, two spectra were recorded with an accumulation time of 2 s and averaged.

The intensity of the symmetric stretching of C-H bond in CH_2_ groups was calculated as integral of the area under the spectrum in the range (2850±15) cm^−1^. This range approximates the bandwidth of the Stokes laser used in CARS microscopy.

### Statistics

All data is expressed as mean ± standard deviation. Statistical analyses to analyze brain tumors (paired t-test or one-way ANOVA followed by Tukey Multiple Comparison test) were performed using Graph Pad Prism 6.0 (Graph Pad Software Inc., La Jolla, CA, USA).

## Results and Discussion

We studied focal brain tumors induced in mice using U87MG human glioblastoma cells, metastasis of human A375 melanoma and of human MCF-7 breast cancer cells as well as human glioblastoma samples to evaluate CARS imaging for label-free delineation of the tumor from the surrounding normal brain parenchyma.

### Localization of primary and secondary brain tumors by label-free CARS imaging

Figure 1A shows the CARS image of an unstained complete horizontal cryosection of a mouse brain bearing a GBM in the hippocampus. The intensity of the CARS signal is represented in grayscale. In our experiments, the C-H molecular vibration was addressed and tissue regions characterized by high lipid content appear brighter in the CARS image. Therefore, white matter tracts can be discerned from gray matter regions. Tumor growth alters the structure and the chemical composition of the tissue, generating specific features in the CARS images. It is known that human GBM and astrocytic tumors have a lower content of total lipids in the fresh tissue weight compared to normal gray and white matter [19]. This feature was already extensively addressed by vibrational spectroscopy in primary [20]–[23] as well as in secondary [24] brain tumors. As a consequence of the lower lipid content, the tumor can be localized in the CARS images: It represents the region with the lowest CARS signal intensity (Fig, 1A, arrow). Figure 1B shows the same tumor at higher magnification and demonstrates that the same kind of intensity contrast also exists at smaller scales, enabling discrimination of a clear interface between the tumor and the surrounding white matter fiber tracts. Brain metastases of breast cancer (Fig. 1C) and of melanoma (Fig. 1D) also displayed a lower CARS signal than the normal nervous tissue. The borders of the tumors were localized with high precision and are in perfect agreement with the corresponding H&E staining (Fig. 1E–H).

In all tumors analyzed (n = 16), the neoplastic tissue was always discerned from the surrounding normal tissue by exploiting the lower CARS signal intensity. Nucleus-rich hippocampal layers (non-tumor tissue) did not display a reduced CARS signal intensity (arrowheads in Fig. 1B/F and 1D/H).

### CARS imaging of tumor infiltrates at cellular resolution

**Figure 2:**
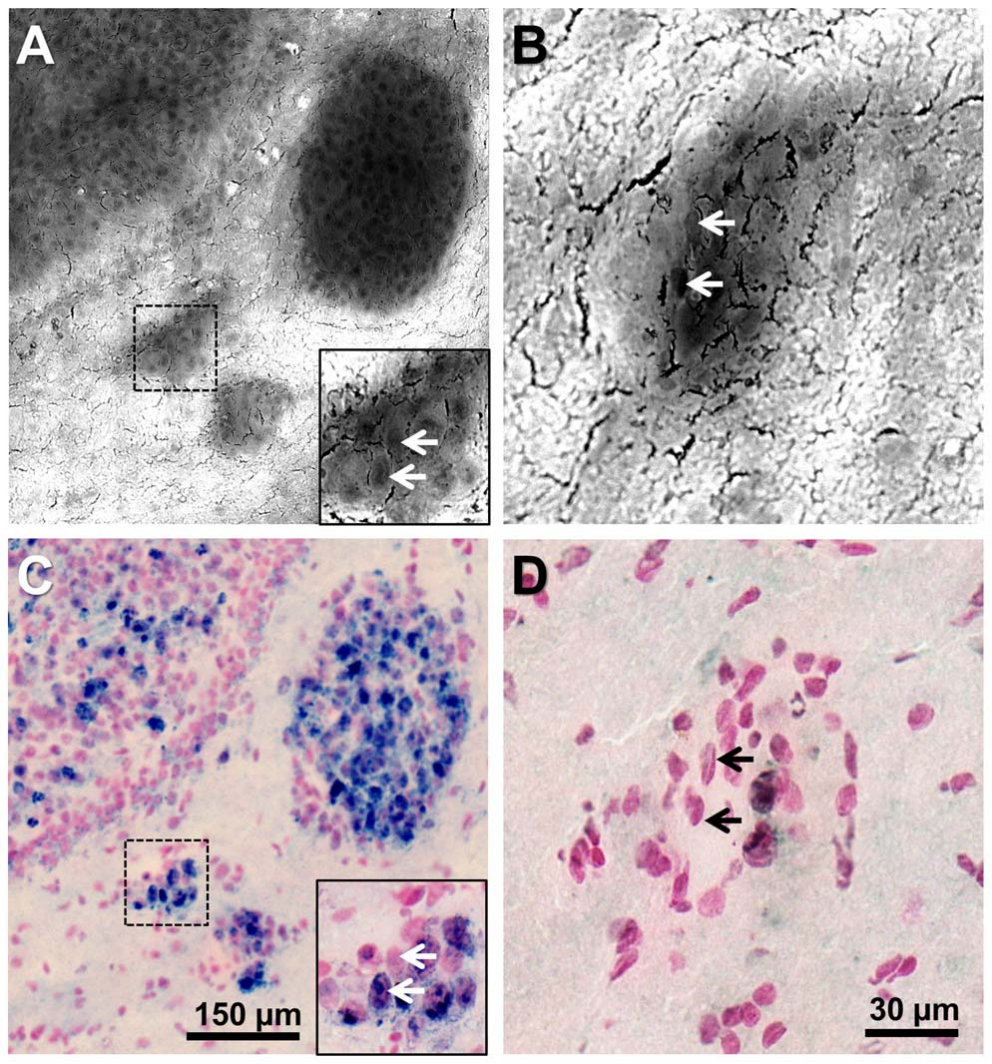
Imaging of the infiltrating tumor margin. **A**: CARS image of a human U87MG glioblastoma in a mouse brain. **B**: CARS image of a separate small glioblastoma island in a mouse brain. **C/D**: Anti-Ki67 immunohistochemical staining corresponding to A/B. In both examples the very same section was used for CARS imaging and for staining.

The lateral resolution of CARS imaging is well below the micrometer (0.6 µm in our system configuration). Therefore, the technique provides cellular resolution and enables the detection of small tumor infiltrates (Fig. 2A) down to a few cells (inset in Fig. 2A, Fig. 2B). At high magnification, single cell nuclei appear as dark structures in the tumor (arrows in inset in Fig. 2A and Fig, 2B). Additionally, non-nuclear parts (i.e. cytoplasmatic or extracellular regions) are darker than the surrounding gray matter. This allows a precise delineation of the border of tumor islands. The size and position of micrometastases detected by CARS imaging matched the histological findings obtained by anti-Ki67 immunohistochemical staining of the same tissue section (Fig. 2C and 2D).

**Figure 3:**
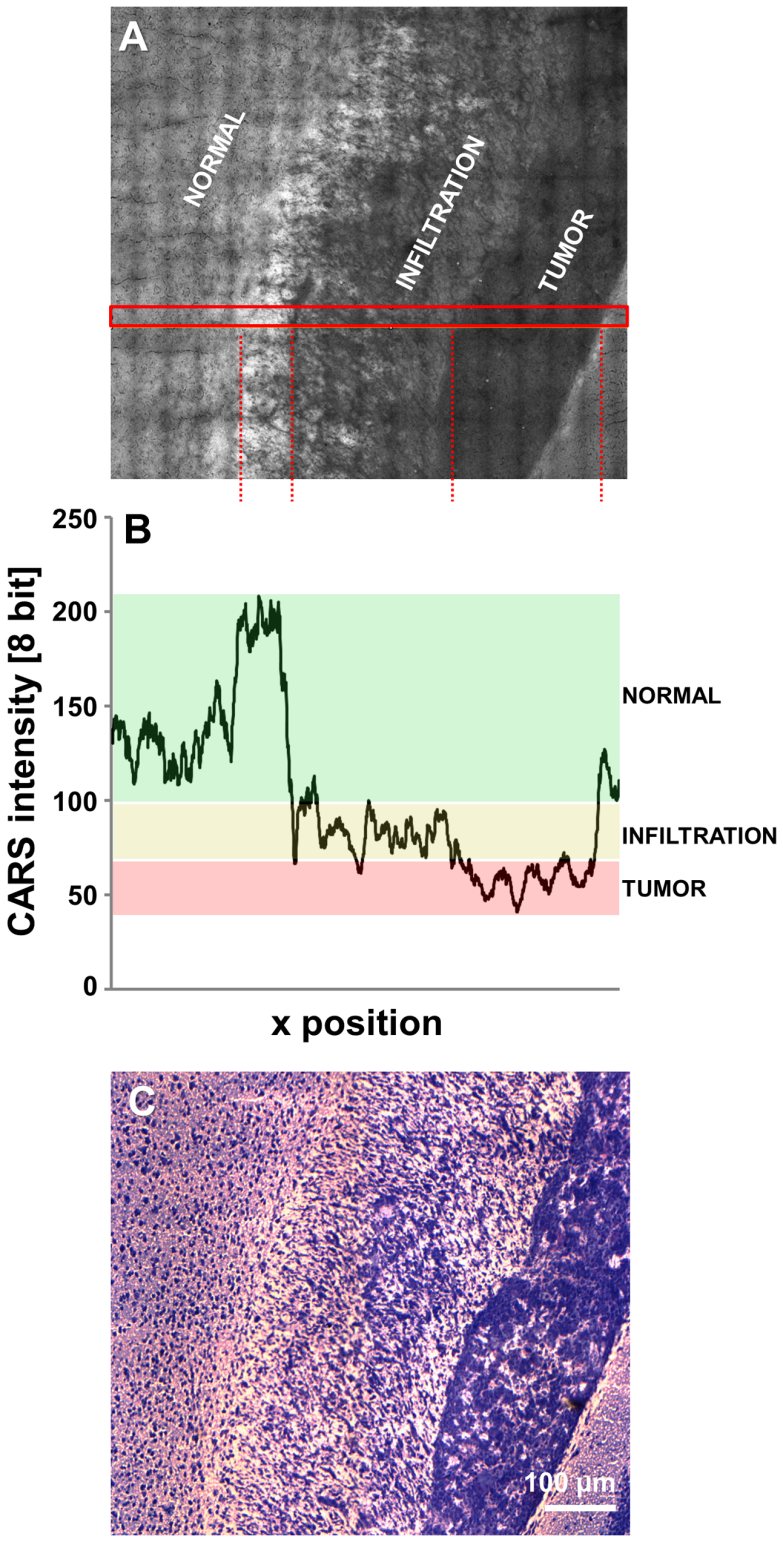
Quantification of the CARS signal intensity of infiltrative tumor areas. **A**: Unprocessed CARS image of a human U87MG glioblastoma in a mouse brain, displaying a solid tumor and a large infiltrative region. **B**: CARS signal intensity along the area indicated in A. The CARS signal intensity range indicative of normal tissue is underlined in green, the intensity range indicative of infiltrative areas in yellow and the intensity range indicative of tumor in red. **C**: H&E staining corresponding to A.

The imaging of a GBM with infiltrative characteristics demonstrated a gradual decline of the CARS signal intensity from normal gray matter via infiltration zone to solid tumor (Fig. 3A, reference H&E staining in Fig. 3C). The intensity of the CARS signal in the area indicated in Fig. 3A is plotted in Fig. 3B and reveals a marked change in the CARS signal intensity along the selected area: Intermediate intensity values are found in the infiltrative zone (average gray value: 85) compared to normal gray matter (average gray value: 126) and tumor (average gray value: 60). This allows the objective identification of the infiltration zone, which is clearly discerned from the surrounding gray matter.

### Quantification of CARS signal reduction

In order to analyze the reduction of the CARS signal intensity, the average pixel intensity (I) of gray matter and of the tumor was determined and the ratio I_tumor_/I_gray_ was calculated. All images were acquired using the same laser parameters and microscope objective, but the photomultiplier gain was adjusted to optimize the dynamic range of each image. Because the ratio I_tumor_/I_gray_ is independent from the photomultiplier gain this value is a robust parameter for the comparison of tumor-induced changes of CARS signal intensity among different samples.

**Figure 4:**
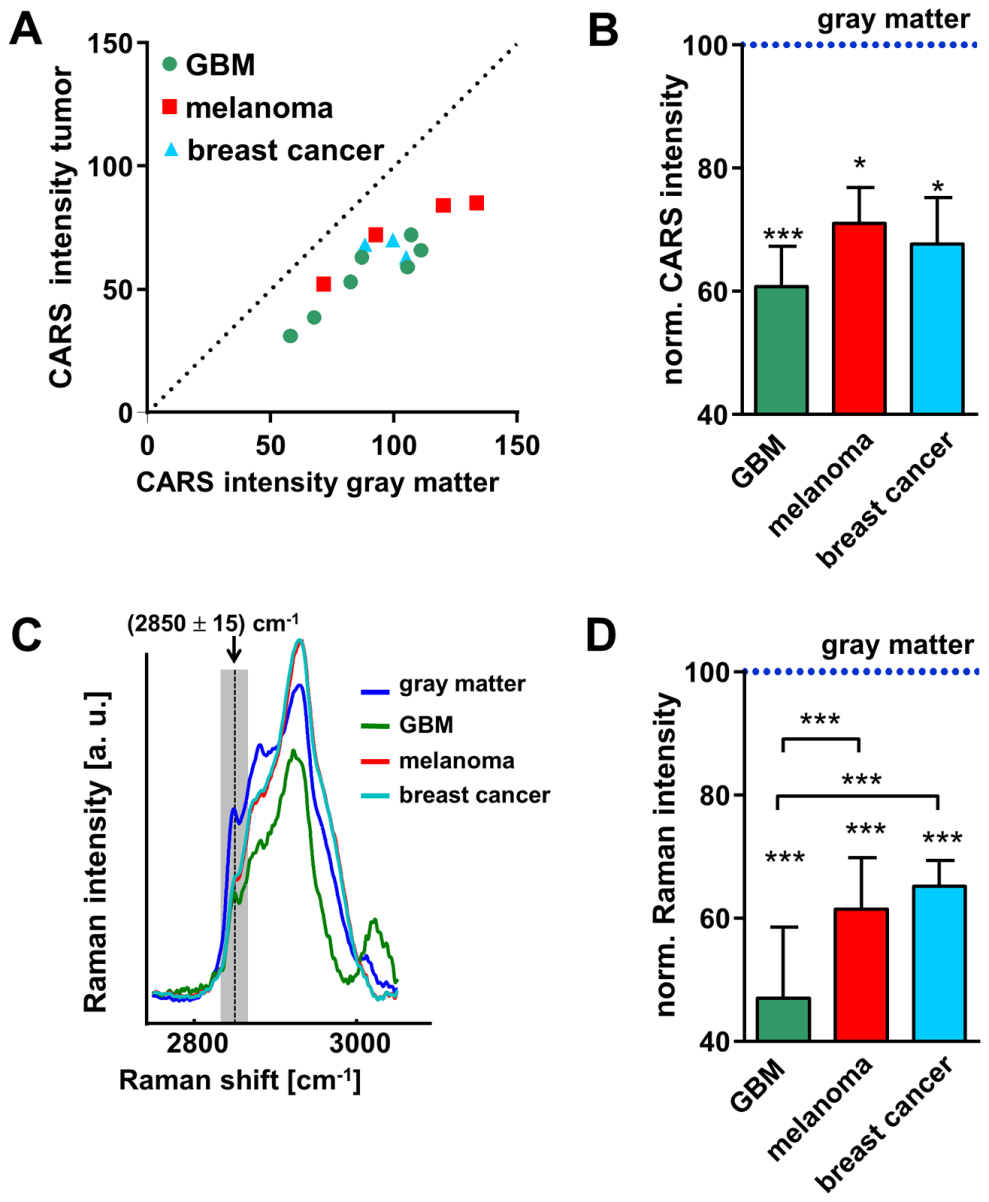
Quantification of the CARS signal intensity and lipid-related Raman band intensity. **A**: Dot plot showing the CARS signal intensity in normal gray matter vs. the intensity of the CARS signal in the neoplastic tissue for each sample. **B**: CARS signal intensities of tumors normalized to the respective intensities in gray matter. **C**: Average Raman spectra of normal gray matter, glioblastoma, melanoma, and breast cancer metastases. **D**: Intensity of the Raman band generated by symmetric stretching of the C-H bonds in CH_2_ groups at 2850 cm^−1^, calculated as integral in the range (2850±15) cm^−1^. Values were normalized to values of gray matter. **B/D**: Bars represent mean ± SD, GBM n = 8; melanoma metastases n = 4; breast cancer metastases n = 4; * significant difference vs. gray matter: P<0.05; *** significant difference vs. gray matter or as indicated: P<0.001.

The CARS signal intensity within the tumor was lower than within gray matter in all samples that were investigated (Fig. 4A, P<0.001). The decrease was more pronounced in GBM than in both types of metastases (Fig. 4B). On average, the CARS signal intensity in GBM was reduced to 61% with respect to normal gray matter, to 71% in melanoma metastases and to 68% in brain metastases of breast cancer.

CARS imaging tuned to resonantly excite the C-H bond vibrations in CH_2_ groups displays the distribution of lipids in tissues, but the CARS signal has an additional, non-resonant component that mainly carries topological information [25]. As a consequence, it is not possible to directly relate the CARS signal intensity to the content of lipids within the tissue, and the ratio I_tumor_/I_gray_ to the reduction of lipid content. Therefore, Raman spectroscopy, which provides quantitative information about the lipid content in the tissue [26]–[28], was performed to alleviate any uncertainty about the lipid contribution to the observed CARS signals.

Multiple Raman spectra were acquired within the tumor and the gray matter. The average spectra of different tumor entities are shown in Fig. 4C and demonstrate that the three types of tumors are characterized by different spectral profiles in the range of C–H stretching vibrations between 2800 and 3000 cm^−1^. The quantification of the intensity of the Raman band at 2850 cm^−1^ - the one addressed in CARS imaging - confirmed the findings of the CARS signal measurements: The intensity reduction was significantly more pronounced in GBM compared to both types of metastases (P<0.001). The intensity of this Raman band was reduced to 47% in GBM, to 61% in melanoma metastases, and to 65% in breast cancer metastases compared to normal gray matter (Fig. 4D).

CARS intensity and Raman band intensity values cannot be directly compared because the two processes are of inherently different nature [6], but the good agreement of results obtained by these two methods supports the possibility of extracting useful data from the CARS images without removing the nonresonant background and despite morphological sample artifacts.

The alterations induced by brain tumor growth in the tissue lipids are complex processes. The direction of lipid changes (decrease or increase) as well as its extent strongly depends on the type and fraction of brain lipids, e.g. gangliosides and phospholipids [29]. Docosahexaenoic acid and phospholipids were significantly reduced to ∼50% in human glioma, while the proportion of linoleic acid among total lipids was increased compared to non-malignant brain tissue [30]. CARS imaging based on excitation of a single Raman band cannot deliver information about changes in relative quantities of the different lipid species. Multiplex CARS [11] or high spectral resolution CARS [31] could alleviate this short-coming and address more than one Raman band, allowing the simultaneous analysis of different lipid species and/or other biochemical tissue components. The differences seen in the region of C-H stretching vibrations in the Raman spectra of GBM and metastases (Fig. 4C) suggest that this might even allow the distinction of primary and secondary brain tumors that was not attained through addressing the band at 2850 cm^−1^ only. The additional analysis of the Raman band at 2930 cm^−1^ could enable discrimination of metastases and glioblastoma, as this band is more intense in the spectra of metastasis and less intense in the one of glioblastoma. Special laser systems are available that allow selective but simultaneous CARS excitation of the Raman bands at 2850 and 2930 cm^−1^, as well as biochemical tissue characterization by automated data analysis [9].

Previous reports [9], [10], [14] have already exemplarily shown that brain tumors are characterized by a decreased CARS signal intensity. Here, we extended the research to different tumor types and finally demonstrated that the decline in the CARS intensity is a reliable parameter to discern the tumor irrespective from its origin and histopathological characteristics from the surrounding healthy parenchyma.

### Tissue properties influencing CARS signal intensity

**Figure 5:**
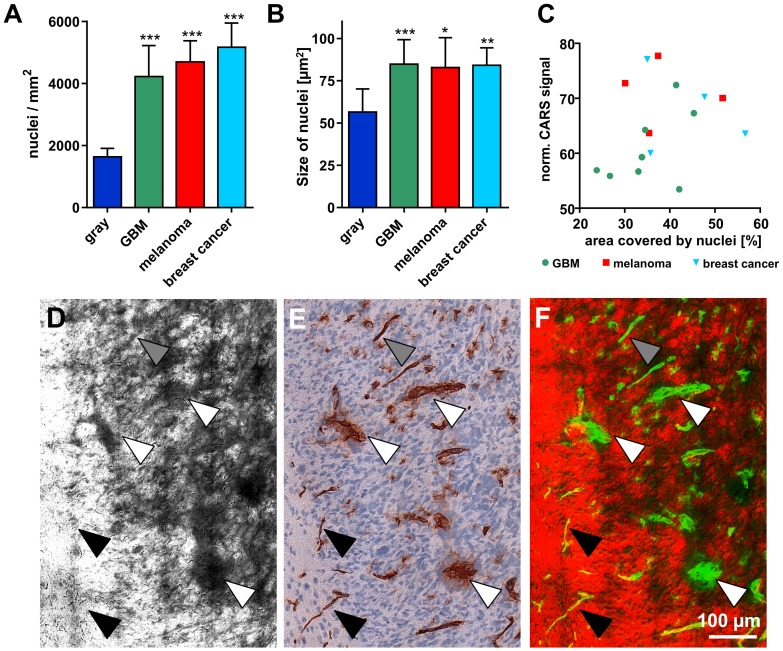
Tumor-induced changes influence the CARS signal intensity. **A/B**: Number of nuclei/mm^2^ representing cellular density (A) and size of single nuclei (B) within normal gray matter and different experimental tumors in a mouse brain. Bars represent mean ± SD, GBM n = 8; melanoma metastases n = 4; breast cancer metastases n = 4; *** significant difference vs. normal gray matter: P<0.001. **C**: Dot plot showing the total area occupied by cell nuclei vs. the normalized CARS signal intensity of the respective tumor. **D**: CARS image of an experimental GBM induced in a mouse brain. **E**: Anti-CD31 staining of a consecutive section of the one shown in D. **F**: Overlay of CARS (red) and anti-CD31 (in false color: green). Coarse blood vessels (white arrowheads) and fine blood vessels (gray arrowheads) are detected in the CARS image. Very fine (normal) blood vessels do not cause any alterations of the CARS signal (black arrowheads).

In previous studies on CARS imaging of brain tumors it was assumed that the contrast generated by tumors in the CARS image arises from the destruction and displacement of normal lipid-rich brain tissue by the comparatively highly cellular and thus lipid-deficient tumor [9]. Nuclei appear darker in the CARS images (as pointed out in the description of Fig. 2) because they are devoid of lipids. Therefore, areas with increased cellularity or enlarged nuclei are expected to generate a lower average CARS signal. Until now it had not been evaluated in detail whether the decreased CARS signal intensity is a direct consequence of the tumor's high cellularity caused by varying ratios of lipid-poor nuclei and lipid-rich cytosol [14]. Hence, the cellular density and the size of single nuclei within the tumors were assessed to calculate the total cross-sectional area covered by nuclei. As expected, the nuclear density was strongly increased in all tumors investigated (Fig. 5A) and a marked increase of nuclear dimension was detected (Fig. 5B). The cross-sectional area that is covered by nuclei was significantly increased in tumors (GBM: 35.1%; melanoma metastases: 38.7%; breast cancer metastases: 43.8%) compared to normal gray matter (9.3%). Interestingly, we did not find a correlation between average CARS signal intensity of the tumor and its total cross-sectional area of cell nuclei (Fig. 5C).

An obvious difference between normal and neoplastic tissue is the proliferative activity of tumor cells. Among the tumors investigated, we found different patterns of proliferation. An uneven, patchy distribution of Ki67-positive and negative nuclei was seen in some parts (Fig. 2C, upper left), while in other tumor parts almost all nuclei were positive for Ki67 (Fig. 2C, large tumor island). By comparing the CARS image and anti-Ki67 immunohistochemical staining of the very same cryosection at a cellular level, we found that the CARS signal intensity is likewise reduced within the whole micrometastasis (Fig. 2A inset and Fig. 2B). Furthermore, the proliferation index for each tumor was determined, but did not correlate with the calculated CARS signal reduction (Fig. S1).

Tumor growth and development is accompanied by tumorigenic transformation of brain microvessels towards a coarse and irregular phenotype. The comparison of the CARS image of a tumor infiltrating white matter (Fig. 5D) and the corresponding anti-CD31 immunohistochemical staining (Fig. 5E) revealed areas of reduced CARS signal that match the positions of coarse and highly transformed blood vessels (white arrows) as well as of fine, almost normal blood vessels (gray arrows). The structure of the very fine blood vessel network of normal brain tissue cannot be retrieved in the CARS image (black arrows). Figure 5F shows the overlay of CARS image (red) and CD31-positive blood vessels (green) in false colors. This suggests that tumors with a strongly altered, coarse blood vessel network would exhibit a lower overall CARS signal intensity. The analysis of the microvessel density and micromorphology of all tumors revealed that the microvessel network was transformed in a similar degree towards a coarse and irregular phenotype in all tumors investigated (vascular index: GBM: 2.71; melanoma metastases: 2.75; breast cancer metastases: 2.65). The microvessel density was comparable in GBM (154.9/mm^2^) and metastases of melanoma (153.6/mm^2^), but lower in metastases of breast cancer (87.9/mm^2^). However, this did not correlate to the extent of CARS signal decline within the respective tumors (lFig. S2).

In this study, we found that tumor-induced changes of the cyto- and tissue architecture, e.g. nuclear size and density as well as blood vessel transformation, all contribute to the CARS signal reduction. Nevertheless, none of the aforementioned histopathological parameters alone provides a correlation to the decrease of CARS signal intensity. Apparently, the quantity of lipid-rich components in tumor cells is intrinsically different compared to gray matter. This is supported by the following observations: i) the CARS signal intensity in tumors is lower than in other nuclear-rich structures, i.e. the normal hippocampus (as already noted in Figure 1 B/F and D/H, see arrowheads), and ii) cytoplasmatic regions of tumor cells display a reduced CARS signal intensity compared to the surrounding gray matter (see Figure 2 A/B). Therefore, the observed decline in CARS signal intensity is not only related to increased presence of lipid-poor structures, but seems to present – in our experimental setting and the human brain tumor tissues investigated so far – a common biochemical feature of tumor tissue. This observation might be attributable to an altered metabolism and signaling in tumor cells [32].

### CARS imaging of human GBM

**Figure 6:**
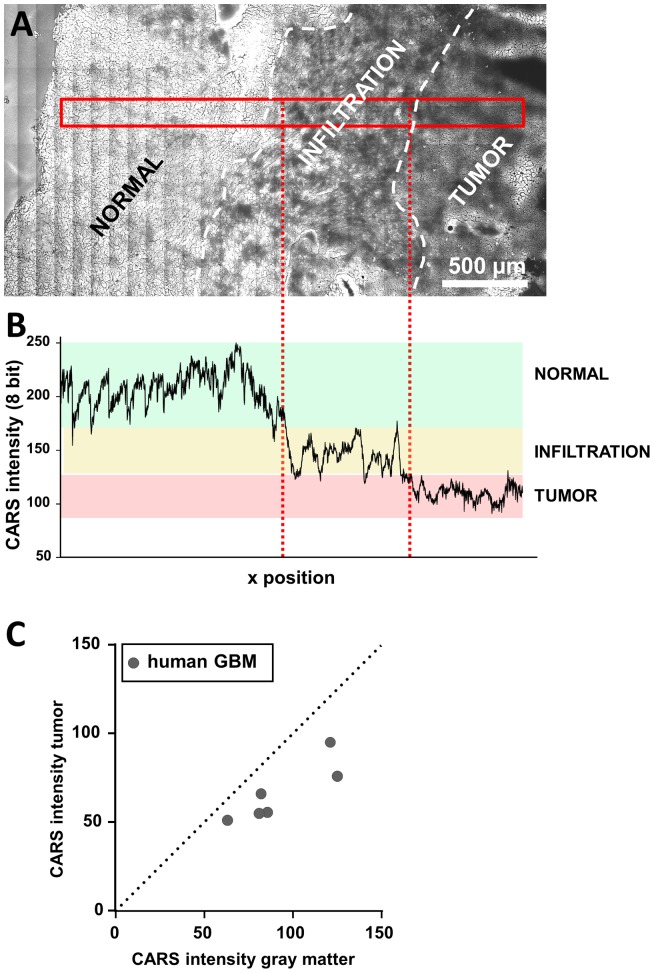
Quantification of the CARS signal in human GBM. **A**: Unprocessed CARS image of a cryosection of a human GBM specimen obtained during routine surgery. The CARS image displays the margin of a solid tumor and an infiltrative region. **B**: CARS signal intensity along the area indicated in panel A. The range of CARS signal intensity of normal tissue is underlined in green, of infiltrative areas in yellow, and of tumor in red, respectively. **C**: Dot plot showing the CARS signal intensity in normal gray matter vs. the intensity of the CARS signal in human GBM for each sample.

Finally, we investigated human glioblastoma samples obtained during routine biopsy by CARS microscopy. The CARS image of a cryosection is shown in Figure 6A and confirms the findings obtained in the mouse model. The tumor is characterized by CARS signal intensities lower than the surrounding normal tissue. The analysis of the CARS signal intensity within the sample was sufficient to discern normal tissue, the infiltration zone, and the tumor itself (Fig. 6B). Similar to the results reported for the mouse tumor in Fig. 3, the CARS intensities of tumor, infiltration zone and normal tissue fall in separated ranges. Quantification revealed a reduced CARS signal intensity within the tumor compared to adjacent almost normal brain tissue in all samples of human GBM investigated (Fig. 6C). On average, it was decreased to 72.2±8.8% (n = 6, P<0.005). CARS microscopy of human primary brain tumors delivered information on tumor localization and enabled tumor border identification by exclusively exploiting the chemical contrast derived from lipid distribution in a totally label-free manner.

During resection of brain tumors tumor border detection is critical. This study indicates CARS imaging for reliable brain tumor delineation and detection of infiltration zones or small tumor island within surrounding brain parenchyma. In those cases the area of interest contains tumor tissue and gray (or white) matter. Therefore, absolute CARS intensities are not required and tissue analysis using the parameter Itumor/Igray can deliver stable and reliable diagnostic information. This opens new perspective for in-situ tumor delineation and fast intraoperative pathology. Additionally, the morphological content of the CARS image can be combined with other multimodal modalities such as TPEF and SHG can deliver information about e. g. cellularity or bloodvessels [14], [33] that can help to identify either tumor or normal tissue. In future clinical intraoperative applications the combination of standardized acquisition parameters and normalization using a pure non-resonant image [34] will enable to directly classify tissue types based on the CARS signal intensity. CARS imaging has the potential to be used before removal of suspicious tissue giving a direct feedback about tissue status to the neurosurgeon.

The extent of resection and residual volume are significantly associated with survival and recurrence in newly diagnosed glioblastoma [35] and both primary and secondary [36] brain tumors may display infiltrative growth characteristics. Therefore, an accurate intraoperative tumor delineation and detection of infiltrates enabling extensive tumor resection is expected to result in improved patient outcomes. CARS microscopy constitutes a fast imaging technique: The image acquisition rate ranges from few Hz in our experimental setup to more than 20 Hz. [37], [38] Therefore, the acquisition speed is compatible with in vivo applications. Further technical developments will pave the way to a clinical, intraoperative application. It has already been shown that CARS technology can be implemented in miniaturized exoscopes [39] or an endoscopic setup [40], [41], which allows video-rate CARS imaging of nervous tissue in vivo [42]. Recent research demonstrated that a scan rate of seven frames per second can be achieved also with endoscopic systems [43]. This imaging speed is well compatible with an intraoperative visualization of brain tumor borders for the neurosurgeon during ongoing surgery. Acquisition times of the large overview images presented in this study provide an impression of the time required for retrospective neuropathological analysis of tissue sections using CARS microscopy. The final histopathology is performed after surgery on tissue sections of the removed tumor. Immunohistochemical analyses that demand several hours are required for exact diagnosis and tumor grading.

The combination of CARS imaging in a setup with second harmonic generation and endogenous two-photon excited fluorescence for extended neuropathology is possible and could complement diagnosis. The multimodal information of the distribution of fibrillar collagen and endogenous fluorophores combined with CARS imaging provides additional and more detailed morphochemical information of brain [33], [44]. This information can be retrieved in situ or retrospectively on unprocessed bulk tumor samples or tissue sections without the need to perform time consuming and expensive immunohistochemical stainings.

In conclusion, CARS microscopy has the potential to become a technology routinely used in neuropathology for assessment of brain tumors. For diagnostic purposes, the possibility to identify different types of tumors by addressing their biochemical characteristics, joined with a fine spatial resolution, gives a significant advantage to CARS microscopy over conventional imaging techniques based on magnetic resonance and intraoperative fluorescent dyes. When implemented in an endoscopic setup, CARS microscopy could be used intraoperatively, providing the surgeon with a rapid and non-invasive tool for precise identification of boundaries and infiltrations of both gliomas and metastases.

## Supporting Information

Figure S1 **The decline of CARS signal intensity is not related to the tumor's proliferation rate.** Dot plot showing the proliferation index of each tumor investigated vs. the normalized CARS signal intensity of the respective tumor.
(TIF)

Figure S2 **The decline of CARS signal intensity is not related to the tumor's microvessel density.** Dot plot showing the microvessel density of each tumor investigated vs. the normalized CARS signal intensity of the respective tumor.
(TIF)
