# Supplementary figures and images for: Label-Free Delineation of Brain Tumors by Coherent Anti-Stokes Raman Scattering Microscopy in an Orthotopic Mouse Model and Human Glioblastoma
Source: PLoS One. 2014 Sep 8;9(9):e107115. doi: 10.1371/journal.pone.0107115 (PMC4159970; doi:10.1371/journal.pone.0107115)

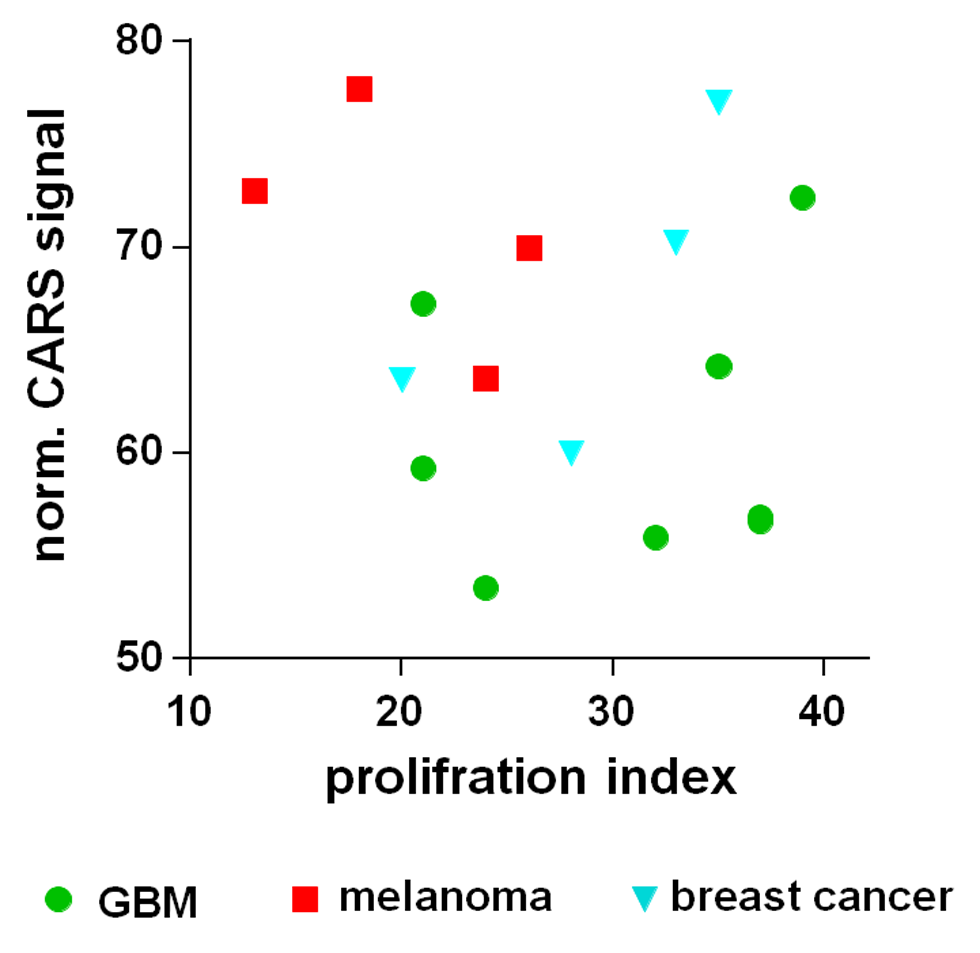

Supplement: Figure S1 — The decline of CARS signal intensity is not related to the tumor's proliferation rate. Dot plot showing the proliferation index of each tumor investigated vs. the normalized CARS signal intensity of the respective tumor. (TIF) [file pone.0107115.s001.tif]

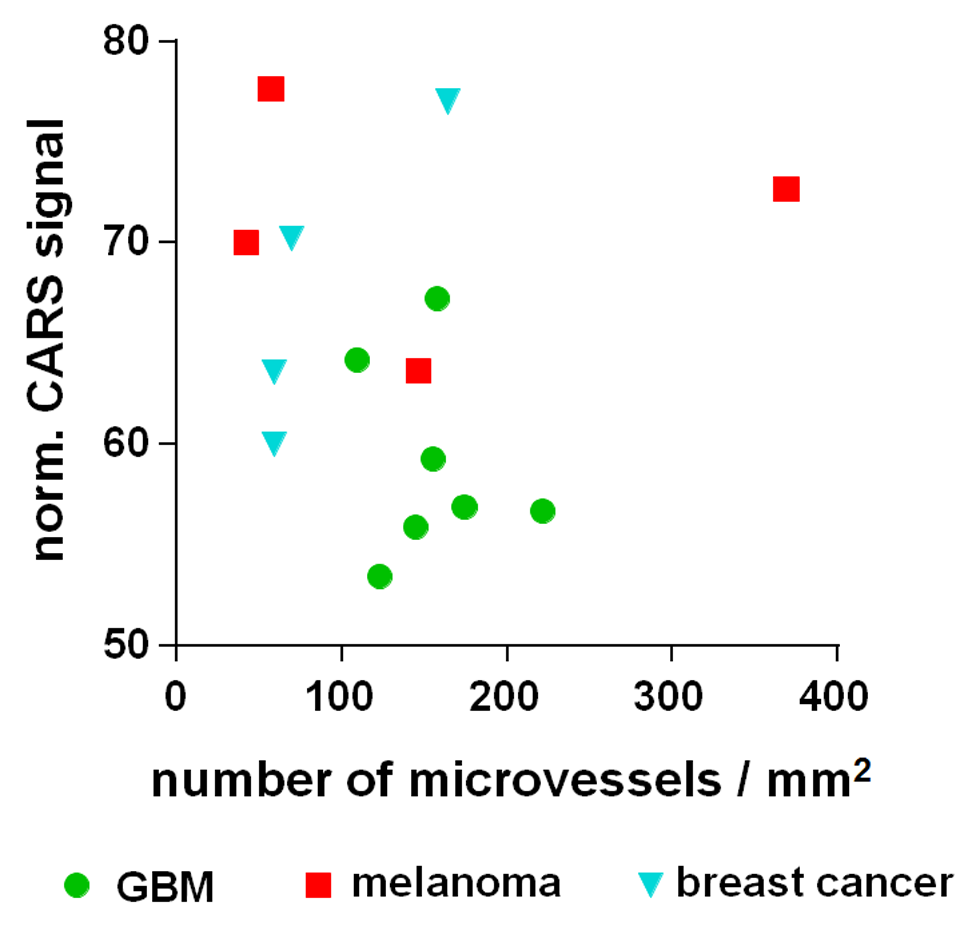

Supplement: Figure S2 — The decline of CARS signal intensity is not related to the tumor's microvessel density. Dot plot showing the microvessel density of each tumor investigated vs. the normalized CARS signal intensity of the respective tumor. (TIF) [file pone.0107115.s002.tif]
